# Upper versus lower body resistance exercise with elastic bands: effects on cognitive and physical function of institutionalized older adults

**DOI:** 10.1007/s41999-022-00616-6

**Published:** 2022-02-12

**Authors:** Miguel A. Sanchez-Lastra, Silvia Varela, José M. Cancela, Carlos Ayán

**Affiliations:** 1 Universidade de Vigo, Departamento de Didácticas Especiais, Facultade de Ciencias da Educación e do Deporte, Campus A Xunqueira s/n, 36005 Pontevedra, Spain; 2 Universidade de Vigo, Grupo de Investigación HealthyFit, Departamento de Didácticas Especiais, Facultade de Ciencias da Educación e do Deporte, Campus A Xunqueira s/n, 36005, Pontevedra, Spain; 3 Instituto de Investigación Sanitaria Galicia Sur (IIS Galicia Sur) Sergas-UVIGO, Vigo, Spain; 4 Universidade de Vigo, Grupo de Investigación Wellness and Movement, Departamento de Didácticas Especiais, Facultade de Ciencias da Educación e do Deporte, Campus A Xunqueira s/n, 36005 Pontevedra, Spain

**Keywords:** Exercise, Frail older people, Institutionalization, Cognitive function, Physical function

## Abstract

**Purpose:**

To compare the effects of upper versus the lower-body resistance exercise on cognitive and physical functions of institutionalized older people.

**Methods:**

This was a non-randomized multi-center comparative and crossover study (clincialtrials.gov code NCT03831373). Two experimental groups performed a 12-week intervention of resistance exercises with low-intensity elastic bands, one program focused on exercises of the upper body (*n* = 20, mean age 87.6 ± 6.4 years, 75% women) and the other on the lower body (*n* = 29, mean age 81.4 ± 7.7 years, 55% women). Following 12 weeks of detraining, the groups performed the other intervention. After another 12 weeks, a follow-up assessment was carried. The control group (*n* = 19, mean age 81.3 ± 9.5, 68% women) performed a full body stretching exercise program in both phases. Before and after each period, cognitive and physical function was assessed by standardized test (Mini-Mental State Examination, Trail Making test and Phototest; Timed Up and Go, Back Scratch, Chair Sit and Reach and had grip strength, respectively). Intention-to-treat and per-protocol analyses were carried.

**Results:**

After the first intervention, significant improvements (*p* < 0.05) were observed in the cognitive function in both experimental groups, and in the hand grip strength in the group that performed lower-body exercise. After the second phase, all groups showed improvements in lower-body and shoulder flexibility and a significant worsening in hand grip strength. The lower-body exercise group showed a worsening in cognitive function, and the upper-body group in functional mobility and dynamic balance.

**Conclusions:**

Resistance exercise with elastic bands showed beneficial effects on cognitive function and functional independence in institutionalized older adults. While upper body exercises seemed to be more effective on cognitive function, lower limb exercises showed better results on physical function parameters.

**Supplementary Information:**

The online version contains supplementary material available at 10.1007/s41999-022-00616-6.

## Key summary points

**Aim:**

We analyzed the differential effects of two resistance exercise programs based on the upper versus the lower body on cognitive and physical functions of institutionalized older people.

**Findings:**

After the first intervention, significant improvements were observed in the cognitive function in both experimental groups, and in the hand grip strength in the group that performed lower-body exercise. After the second phase, all groups showed improvements in lower-body and shoulder flexibility and a significant worsening in hand grip strength. The lower-body exercise group showed a worsening in cognitive function, and the upper-body group in functional mobility and dynamic balance.

**Message:**

Resistance exercise with elastic bands is safe in institutionalized older people. Upper body exercises seemed to be more effective on cognitive function, while lower limb exercises showed better results on physical function parameters.

**Supplementary Information:**

The online version contains supplementary material available at 10.1007/s41999-022-00616-6.

## Introduction

During ageing, a deterioration of the physical function is produced by loss of functionality of different organs and systems, as well as of muscular mass and strength [1]. In the cognitive function, ageing is characterized by a reduction of brain tissue and functionality, which induces lower processing speed, working memory or episodic memory, among other functions [2]. This process as a whole derives in frailty, lower functional independence for daily life tasks and lower quality of life [3].

Physical exercise is essential in slowing down the impact and consequences of the ageing process in the organism [3]. In particular, strong evidence indicates that resistance exercise has numerous benefits at the physical and functional level of this population (e.g., maintenance and improvement of strength and muscle mass, functional mobility, or prevention of falls) and may also be beneficial at the cognitive level (e.g., for attention, memory and verbal fluency) [4], both in people with early and late stages of sarcopenia and frailty[5].These benefits have been observed in both older people living independently and in people living in residential homes, although considerably less so in the latter group.

Resistance exercise can also be performed with elastic bands, which is a low-cost material of great interest, since elastic bands allow great adaptability and have been shown to be useful in achieving the beneficial effects. More precisely, previous studies in older residents using this material have reported benefits on a number of fitness dimensions, including strength [6–9] agility, balance and functional mobility [7, 9–11], flexibility and aerobic endurance and also in certain metabolic biomarkers (i.e., blood lipids and glucose levels) [9] and self-perceived health [11], which indicates the strong positive impact that this training modality can have on this population. In addition, performing exercises with elastic bands have led to important improvements not only in mobility outcomes but also in cognitive function [7, 11, 12]. These two factors experience a considerable decline in institutionalized people, and strongly affect their functional autonomy and independence [4].

Mobility limitations have a high prevalence among older adults living in residential homes [13]. Despite the benefits of exercise programs for people with and without reduced mobility, older people living in residential homes tend to higher prevalence of physical inactivity and lower levels of participation in the centers' exercise programs [14, 15]. Self-perceived lack of balance, health risks derived from exercise and fear of falls are some of the most cited barriers for exercise participation in this population. Therefore, offering exercise programs that make the residents feel safe and belief in their capabilities and that can lead to the desired outcomes (e.g., improved functional independence), has the potential to increase participation and the achievement of the outcomes [14]. One strategy to increase participation and ultimately functional independence of the residents could be to offer exercise programs focused on one area of the body (i.e., the upper or lower body), so that residents that feel insecure to engage in exercise programs with the other area perceive the programs as more feasible and safer. To this purpose, it is important to analyze if participating in programs only with the upper or lower body can bring benefits in general health-related aspects such as general functionality and cognitive function, and if there are differences in the effects from focusing on each area. Nevertheless, current evidence is mainly limited to full body programs or programs performed only with one area of the body. Recent clinical trials have examined the effects of strength training focused on the lower or upper body, suggesting that it may lead to improvements in strength in the body area that was not involved in performing the exercises [16]. In other studies, the effects of performing targeted strength exercise on markers related to muscle mass [17] or cellular stress [18] have also been examined. However, no studies have compared the effects of performing strength exercise programs focused on the upper or the lower body in older adults, neither in those living in the community or in those in residential homes.

Therefore, the aim of this study was to analyze the differential effects of two resistance exercise programs based on the upper versus the lower body on the cognitive and physical function of institutionalized older adults.

## Materials and methods

### Trial design

This was a multi-center comparative and crossover study, with two experimental groups and a control group.

### Participants

A total of 68 participants were recruited in three residential homes of Galicia (Spain); DomusVi Barreiro, DomusVi Cangas and DomusVi Bembrive. The inclusion criteria were as follows: (1) no health problems contraindicated for participating in the exercise programs; (2) no severe mobility problems which could affect the completion of any of the programs; (3) being able to follow autonomously the oral instructions on how to perform the exercises during the sessions given by the session’s instructor while performing the exercises. In contrast, a health condition that could likely prevent physical assessment or exercise performance was considered an exclusion criterion.

The ethics committee of the faculty of Education and Sport Sciences of the University of Vigo approved the study (code 16-1009-17). The board of directors of the residential homes gave their consent after being informed of the experimental procedure. Participants were also informed about the procedure and agreed to take part following the medical approval whereby their health would not be at risk at any point in the entire program. This study was performed in accordance with the Declaration of Helsinki and abiding by the European Guidelines on Good Clinical Research Practice (111/3976/88; 1st July 1991), as well as the existing Spanish legal framework concerning clinical research with human subjects (Royal Decree 561/1993, on clinical trials). The protocol of this study is registered in clincialtrials.gov (code NCT03831373).

### Interventions

Those residents in centers one and two who wished to take part in the study formed the experimental groups one (G1) and two (G2), respectively, and the programs for each group were assigned in a random order. Participants in the third center served as control group. This allocation was decided since it was impossible to conduct three interventions in each center simultaneously, due to a lack of health professionals who could administer and control the exercise program accurately. Both experimental groups performed resistance exercises with low-intensity (yellow color) elastic bands. These bands offer a resistance of 0.8 kg at an elongation of 50%, 1.3 kg at 100%, 1.8 kg at 150%, 2.2 kg at 200% and 2.6 kg at 250% elongation. The intensity of the elastic band was maintained throughout the interventions. In the first phase, experimental group one (G1) first carried out a resistance exercise program focused on the upper body, with a duration of three months. Subsequently, this group carried out a period of de-training, lasting another three months. Then, in the second phase, the group carried out a resistance exercise program focused on the lower body, lasting three months. After the second program, a de-training and follow-up phase was included, which lasted another three months. The experimental group two (G2) first carried out the resistance exercise program focused on the lower-body, followed by the de-training period, the same program for the upper-body and finally the follow-up. The intensity (color) of the elastic band remained unchanged across the intervention. The control group (CG) followed the same timing as the experimental groups and performed an identical flexibility exercise program in both phases, involving upper and lower body stretching exercises. All programs were performed three days per week in 50-min sessions, with all sessions comprised of three separate phases: activation, main part and cool down. A detailed description of the resistance exercise and stretching programs is presented in Supplementary Tables 1 and 2, respectively. All sessions were carried in the centers and were administered and supervised by a physiotherapist.

### Outcomes

Change in mental status: The Spanish version [19] of the Mini-Mental State Examination (MEC) was used, as it has shown adequate validity and reliability levels in older adults to assess spatio-temporal orientation, attention span, focus and recall, mental calculation and language skills, visual-spatial perception and the ability to follow basic instructions. A 1.6–2 point change in the score of the MEC is considered clinically meaningful [20]. The second test was the Trail Making Test (TMT) [21]. Given the difficulty of the participants to complete part B, only part A was administered. This test has shown good correlation with other validated cognitive tests [22]. The last of the tests was the Fototest [23], which was used to assess memory through free and cued recall of images, executive function (i.e. verbal fluency) and denomination (i.e. language). The higher the score, the better cognitive function, with no maximum score. This test has also shown good criterion validity and high reliability. The cognitive function tests were administered by the in-house psychologist, blinded to the group-assignment.

Change in physical status: The Timed Up and Go Test (TUG) [24] was used to assess basic functional mobility and locomotive capacity (dynamic balance). The test consists in standing up from a chair, walking a 3-m distance, going back, and sitting again, under the instruction of performing the task “as quickly as possible”. A stopwatch is used to register the time the participant takes to complete the process. This test has shown great reliability [25] and its results are associated with the risk of falling [26]. It has been suggested that a change of 0.9–1.4 s is of clinical relevance in elderly patients with COPD [27]. The Chair Sit and Reach (CSR) [28] from the Senior Fitness Test battery, was used to assess the flexibility of the posterior musculature of the leg. Sitting on the edge of a chair, keeping one leg fully extended, the participant must try to reach the tip of the foot with both hands simultaneously. Then, the distance between the third finger of the hands and the tip of the foot is measured. This test has shown good reliability and criterion validity [29]. Also from the Senior Fitness Test, the Back Scratch (BS) test was used to assess the mobility of the shoulder. The participant must try to touch both hands at the back, with one arm behind the head and the other from the waist, while standing. This test has been widely used to measure the upper body mobility in previous research [30]. Finally, hand grip strength (HG) was measured with a Saehan hand-held dynamometer, with the person standing and holding the device with the arm fully extended, along the body [31]. Both hands were assessed in two occasions and the highest value was used for the analysis. This outcome has been strongly associated to general strength, bone mineral density, risk of falls, cognitive impairment, depression, frailty, morbidity and mortality [32] and the test has shown great test–retest reliability in older adults [33]. Although no consensus has been reached regarding the clinically meaningful change of in grip strength, values of 5.0 to 6.5 kg have been suggested [32]. The physical function tests were administered by a physical activity and sports scientist.

All assessments were performed by a non-blinded assessor. Baseline assessments (T0) were carried two weeks before the interventions started. After the first intervention of 12 weeks, another assessment was done (T1). Following another 12 weeks, the third assessment (T2) was done before the second intervention. After 12 weeks of the second intervention, the fourth assessment was carried (T3). Following another 12 weeks of no intervention, a follow-up assessment was done (T4).

### Sample size

Sample size was calculated using G*Power 3.1 software, based on the results from previous studies [34], with confidence level of 0.05, power of 95%, precision of 4, variance of 90, ratio of 1 between groups and a 15% of losses, sample size was estimated in 35 participants per group.

### Statistical methods

The descriptive analysis of the sample was carried out through central tendency measures (mean and standard deviation). The distribution of the variables in the sample was analyzed by visual inspection of histograms and the Shapiro–Wilk test. Because none of the variables showed a normal distribution, non-parametric tests were used to analyze the differences between each moment of assessment within the same group (Wilcoxon rank-sum test) and between groups at each assessment (Kruskal–Wallis test). In addition, moment per group interaction was analyzed using a two-way ANOVA with each outcome as dependent variable and the group and moment of assessment as independent variables. These three tests were carried in two different approaches. First, as intention-to-treat analysis, including all participants that initiated the study. Second, a per-protocol analysis was carried including only those participants attending at least the 70% of the exercise sessions [35]. The effect size (ES) was calculated for each variable and study group between the different assessments using Cohen's *d* in the case of the intention-to-treat due to the sample size per group (*n* ≥ 20 participants) and Hedges' *g* in the analysis per protocol [36]. Effect size was interpreted as trivial (≤ 0.2), small (> 0.2), moderate (> 0.5), large (> 0.8) or very large (> 1.3) [37]. All analyses were performed using Stata Statistical Software version 16 (StataCorp. 2013. College Station, TX: StataCorp LP), considering *p* < 0.05 for statistical significance.

## Results

**Fig. 1:**
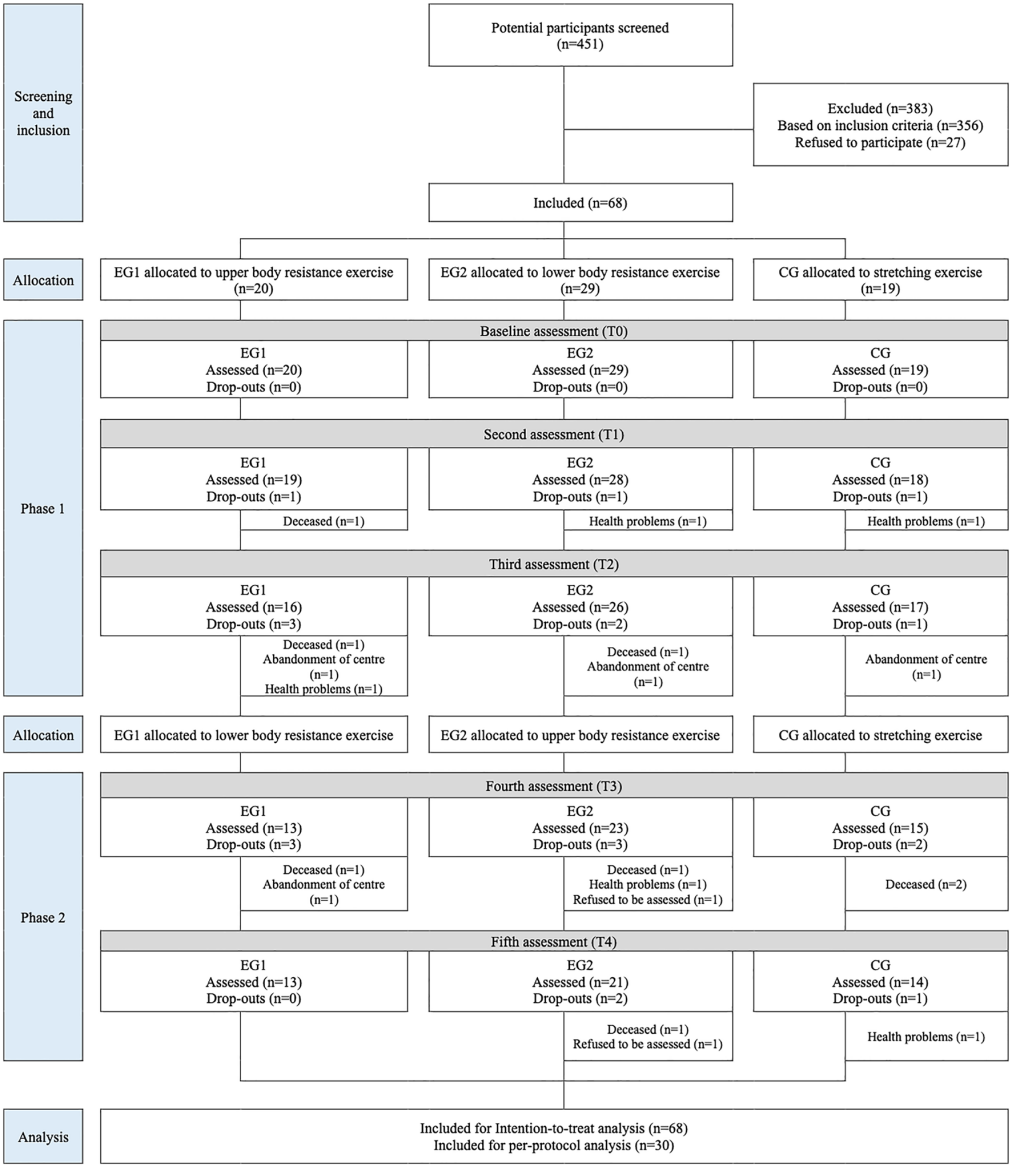
Flow diagram of the progress through the phases of the study

A total of 68 participants (mean age 83.2 ± 8.3 years, 65% women) were included in the study. Of the total, 20 participants were assigned to G1, 29 to G2 and 19 to CG (Fig. 1).

The general characteristics of the sample are summarized in Table 1. At baseline, the groups showed significant differences at baseline in age (*p* = 0.007), and no differences in the other outcomes.

**Table 1:** Baseline characteristics of the sample

Outcome	G1 (*n* = 20)		G2 (*n* = 29)		CG (*n* = 19)	
	Mean ± SD	Range	Mean ± SD	Range	Mean ± SD	Range
Age (years)	87.6 ± 6.4*	72.6, 96.4	81.4 ± 7.7	61.8, 91.9	81.3 ± 9.5	61.0, 93.9
Falls in previous year (no)	0.8 ± 1.2	0.0, 4.0	1.8 ± 2.7	0.0, 11.0	1.6 ± 2.3	0.0, 7.0
MEC (score)	19.2 ± 4.1	10, 27	22.1 ± 4.8	13, 30	18.7 ± 6.9	9, 29
TMT-A (s)	246.8 ± 147.6	86.0, 649.0	314.0 ± 191.5	61.0, 752.0	347.7 ± 152.3	124.0, 590.0
Phototest (score)	24.1 ± 6.8	14.0, 37.0	23.1 ± 9.3	5.0, 41.0	19.3 ± 8.7	11.0, 43.0
TUG (s)	21.9 ± 12.0	8.3, 47.6	18.7 ± 13.5	10.3, 77.0	23.1 ± 19.8	7.0, 96.0
CSR right leg (cm)	− 30.5 ± 10.1	− 44.0, − 8	− 32.3 ± 11.1	− 53.0, 1.5	− 32.8 ± 7.6	− 49.0, − 18.0
CSR left leg (cm)	− 31.2 ± 12.9	− 55.0, − 1.0	− 32.0 ± 9.7	− 53.0, − 14.0	− 30.3 ± 9.2	− 44.0, − 5.0
BS right arm above (cm)	− 29.1 ± 16.8	− 1.0, − 5.0	− 28.9 ± 13.6	− 57.0, − 1.0	− 31.1 ± 13.2	− 54.0, − 2.0
BS left arm above (cm)	− 36.4 ± 17.2	− 70, − 7.0	− 32.4 ± 12.7	− 55.0, 2.0	− 35.9 ± 13.1	− 60.0, − 7.0
HG right (kg)	19.8 ± 6.8	14.0, 40.0	21.7 ± 6.5	9.0, 36.0	21.8 ± 7.3	12.0, 34.0
HG left (kg)	17.9 ± 6.4	8.0, 36.0	20.2 ± 6.3	8.0, 34.0	19.9 ± 7.8	8.0, 34.0

*BS* Back Scratch test, *CG* control group, *CSR* Chair Sit-and-Reach Test, *G1* experimental group 1, *G2* experimental group 2, *HG* hand grip strength, *MEC* Spanish version of the Mini-Mental State-Examination, *TMT-A* Trail Making Test part A, *TUG* timed up and go test

*Significant differences between G1 and G2 (*p* = 0.002), and G1 and G3 (*p* = 0.001)

Results for the intention to treat analysis (Table 2) showed, after the first phase of the intervention (T0 versus T1 measurements), significant improvements in the cognitive function by means of the MEC in G1 after upper-body resistance exercise (*p* = 0.008) and in the TMT-A for both experimental groups (G1 *p* = 0.004, G2 *p* = 0.031). The G2 also improved the hand grip strength (*p* = 0.005) after performing lower-body resistance exercise. After the second phase (T2 vs T3 measurements), all groups showed improvements in lower-body and shoulder flexibility, by means of the CSR (*p* < 0.001) and BS (*p* ≤ 0.038) respectively, and a significant worsening in hand grip strength (*p* ≤ 0.03). The G1 showed a worsening in cognitive function assessed by the MEC after lower-body exercise (*p* = 0.009), and the G2 showed a worsening in functional mobility and dynamic balance, assessed by the TUG, after upper-body resistance exercise (*p* = 0.020). Moment per group interaction analysis revealed significant interactions only for the CSR in T0 versus T1 (*F*[2, 130] = 4.51, *p* = 0.013) and T0 versus T4 comparisons (*F*[8, 325] = 2.10, *p* = 0.035). Percentage of change and effect sizes for each group, outcome and measurement are presented in Supplementary Table 3.

**Table 2:** Results and comparisons across all measurements using intention to treat analysis (G1 *n* = 20, G2 *n* = 29, CG *n* = 19)

Outcome	Measurements (mean ± SD)					Moment × group interaction		
	T0	T1	T2	T3	T4	T0–T1	T2–T3	T0–T4
						*F* (2, 130),* P*	*F* (2, 130), *P*	*F* (8, 325), *P*
MEC (score)								
G1	19.2 ± 4.1	22.1 ± 3.9*	22.1 ± 3.7	20.9 ± 4.2*	19.8 ± 5.2*	1.17, 0.315	*0.19, 0.824*	0.60, 0.782
G2	22.1 ± 4.8	22.0 ± 5.0	21.5 ± 6.2	21.2 ± 6.2	22.1 ± 5.2^a^			
CG	18.7 ± 6.9	18.7 ± 6.1	18.2 ± 6.1	18.6 ± 6.0	17.6 ± 6.4*^a^			
TMT-A (s)								
G1	246.8 ± 147.6	177.4 ± 94.6*^ab^	154.4 ± 56.6^ab^	188.7 ± 60.6^a^	254.8 ± 127.4*^a^	0.25, 0.780	*0.39, 0.680*	0.71, 0.682
G2	314.0 ± 191.4	259.9 ± 142.6*^a^	244.1 ± 140.5^a^	252.1 ± 133.8	266.6 ± 151.14			
CG	347.7 ± 152.3	326.0 ± 161.8^b^	265.7 ± 116.0*^b^	255.9 ± 106.3^a^	276.3 ± 115.9*^a^			
Phototest (score)								
G1	24.1 ± 6.8	24.4 ± 6.1^a^	24.9 ± 5.7	25.0 ± 5.5	21.2 ± 10.7*	0.07, 0.940	*0.03, 0.970*	0.72, 0.676
G2	23.1 ± 9.3	23.1 ± 8.0^b^	23.6 ± 9.5	24.2 ± 8.7	26.5 ± 7.7*^a^			
CG	19.3 ± 8.7	17.4 ± 9.9^ab^	20.3 ± 9.7	21.3 ± 11.4	20.6 ± 10.5^a^			
TUG (s)								
G1	21.9 ± 12.0	22.0 ± 11.4	18.9 ± 6.6	20.5 ± 8.3	16.8 ± 4.6*	0.62, 0.538	0.34, 0.714	0.63, 0.753
G2	18.7 ± 13.5	20.5 ± 19.3	16.7 ± 6.7	20.3 ± 11.5*	20.0 ± 13.2			
CG	23.1 ± 19.8	18.0 ± 9.9	20.1 ± 8.2*	20.8 ± 8.6	22.5 ± 11.6			
CSR (cm)								
G1	− 30.9 ± 11.2	− 37.8 ± 4.5*^ab^	− 26.8 ± 11.4*	− 14.5 ± 12.6* ^ab^	− 15.2 ± 5.4	4.51, 0.013	1.10, 0.340	2.10, 0.035
G2	− 32.1 ± 9.8	− 29.7 ± 10.3^a^	− 25.0 ± 10.8*	− 18.4 ± 7.5* ^a^	− 12.9 ± 6.7*			
CG	− 31.6 ± 8.1	− 26.7 ± 9.6*^b^	− 25.1 ± 10.8	− 18.2 ± 7.4* ^b^	− 13.5 ± 8.0*			
BS (cm)								
G1	− 32.8 ± 16.4	− 29.7 ± 13.6	− 23.9 ± 10.3*	− 19.6 ± 9.0*	− 16.0 ± 6.4*	0.24, 0.788	1.16, 0.316	0.52, 0.839
G2	− 30.7 ± 12.6	− 31.5 ± 12.7	− 29.6 ± 12.1	− 19.3 ± 10.1*	− 17.2 ± 5.2*			
CG	− 33.5 ± 13.0	− 33.0 ± 13.7	− 30.9 ± 13.4	− 20.0 ± 8.9*	− 16.2 ± 5.0			
HG (kg)								
G1	18.9 ± 6.4	19.6 ± 4.4^a^	18.6 ± 3.5	12.8 ± 2.8*	8.5 ± 2.1*^ab^	0.69, 0.503	0.98, 0.38	0.62, 0.760
G2	20.9 ± 6.0	22.9 ± 5.6*^a^	21.4 ± 6.4*	14.2 ± 3.4*	10.8 ± 2.7*^a^			
CG	20.8 ± 7.4	19.9 ± 6.0	19.3 ± 6.0*	14.8 ± 4.3*	11.0 ± 2.5*^b^			

*BS* Back Scratch test, *CG* control group, *CSR* Chair Sit-and-Reach Test, *G1* experimental group 1, *G2* experimental group 2, *HG* Hand Grip strength, *MEC* Spanish version of the Mini-Mental State-Examination, *TMT-A* Trail Making Test part A, *TUG* Timed Up and Go Test. Measurements are T0: Baseline, T1: After first exercise program, T2: after wash-out period; T3: after second exercise program, T4: after the end of follow-up

*Significant differences (*p* < 0.05) with previous moment of assessment for the same group*Significant differences (*p* < 0.05) with previous moment of assessment for the same group

^a^ or ^b^ Significant differences between groups with the same upper script letter in the moment of assessment

The results for the analysis per protocol showed a similar pattern (Supplementary Tables 4 and 5 and Supplementary Figs. 1 and 2), although effect sizes were generally accentuated (Supplementary Table 6).

No harms or adverse events derived from the interventions occurred during the study.

## Discussion

The aim of the study was to investigate the different effects of two resistance training programs using elastic bands, focused on the upper versus the lower body, on physical and cognitive function of institutionalized older adults. Generally, despite the existing evidence on the benefits of strength training in older people, only a few studies have analyzed the effects of resistance exercise programs using elastic bands in the institutionalized population.

One of the main findings was that, after the first three months of the programs, improvements were observed in different domains of cognitive function. In particular, a significant improvement in the global cognitive function following the upper body program was shown. This result is coherent with the findings of a recent review [38], which reported that participating in resistance training programs can lead to improvements in global cognitive function in both cognitively impaired and cognitively healthy older adults. Nevertheless, while this review did not report the possibility of enhancing the specific domain of attention, we found significant improvements in this domain, assessed by means of the TMT-A, following both the upper and the lower body programs. Regarding the domains assessed by the Phototest, memory and verbal fluency, no improvements were found. Previous evidence is unconclusive in respect to the effects of strength training programs in these domains. For example, a previous study [39] showed that both parameters improved with strength training in their investigation, while another study [40] reported no improvements in verbal fluency in a group of cognitively healthy older people. In our study, after the second intervention and with the crossover of programs, a worsening of global cognitive function was detected in the group that performed the lower body strength program, while in the group performing the upper body program, no significant results in any cognitive domain were found. The variation in the results could be explained by uncontrolled confounding factors in the groups, as the results seem more linked to a particular training group than to the program itself. Nevertheless, an important aspect to highlight is that the two groups that performed resistance exercise maintained the global levels of cognition, assessed by means of the MEC test, throughout the duration of the study (from T0 to T4), whereas the control group showed a significant deterioration. This result suggests that both training programs, independently of the order in which they were administered, could be protective and reduce the natural progression of cognitive impairment, which goes in line with previous research [4], while stretching exercise may not be useful in the long term. Nevertheless, this result should be interpreted with caution, as the evolution in the TMT-a and Phototest assessments was similar for the three groups. Altogether, more scientific evidence on the effects of strength training programs on cognitive domains is warranted.

Regarding the effect of the training on physical function, after the first intervention, the group performing the upper body program significantly worsened in lower limb flexibility, while the group performing the lower body program significantly improved its hand grip strength. The scarce evidence available on the effectiveness of strength training with elastic bands on flexibility point to a different direction compared to our results [41–43]. For example, a previous study [41] reported that moderate and high intensity strength training were more effective for improving flexibility than low intensity training. Therefore, the low intensity (i.e., low loads) of our program could partially explain the lack of positive results. Additionally, a recent study using elastic bands for strength training [42] conducted a moderate intensity program in which a progression of loads was established through the use of different elastic bands throughout the intervention, which resulted in significant improvements in the CSR after 12 weeks of training. Therefore, another reason for our null results could be the lack of progression in the loads used in the training.

The results of strength measured by manual dynamometry did not show improvements in the group performing the upper body program but did show improvements in the group that performed the lower body program. Previous evidence is also inconclusive in this respect. A study implementing an 8-week program with elastic bands in detrained older adults did not report significant improvements [44]. Similarly, other authors did not detect changes in dynamometry values after a resistance program combining elastic bands with body weight exercises [45]. Some authors have suggested that a specific upper limb program is necessary to obtain improvements in manual dynamometry [45]. Nevertheless, although a specific upper body strength program was performed in our study, it is possible that the stimulus offered by the material used was not sufficient to induce strength improvements [46]. Furthermore, while some evidence indicates that the positive effects of lower limb strength training may not translate into improvements in manual dynamometry data [47], a previous intervention combining upper and lower limb exercises which did show improvements in handgrip strength assessments [42].

Previous evidence regarding dynamic balance is indecisive as well, as studies with similar training proposals have reported contradictory results. More precisely, while a study with a lower limb only program obtained significant improvements in TUG [48], a full-body strength training program did not show improvements in this parameter [42].

At the end of the second intervention (crossover of programs), the experimental groups showed a significant improvement in both flexibility tests which, as previously indicated, was to be expected after strength training [7]. The lack of improvement in dynamic balance could be explained by the significant worsening of strength observed in this second phase since, according to a recent review [49], both aspects could be linked. Although the mechanisms explaining this relationship are not clear, the presence of a certain level of instability during the practice of resistance training could have a positive influence on balance [49]. Due to the characteristics of the program of the present study, which was mainly performed in a seated position, the aforementioned instability condition did not occur.

Finally, it is necessary to highlight a review that showed that upper limb interventions are associated with greater effects on functional independence while lower limb interventions promote general physical function and prevent disability in older adults [50]. Although the results of the present study do not go in accordance with this idea, the lack of studies comparing upper versus lower limb exercise approaches does not allow to draw firm conclusions in this regard.

Altogether, our results are of clinical relevance for two main aspects. First, they indicate that performing strength exercise with elastic bands (an economic and easy-to-use material), focusing on the upper or lower body, can potentially lead to benefits in the cognitive function, especially when performed with the upper body. Second, performing exercise with the lower body can potentially lead to general strength benefits. This is particularly interesting for people with mobility limitations or high risk of falls, especially in the case of institutionalized older adults, and may encourage the personnel of the centers to include more people in the exercise programs. Additionally, this study may serve as a basis for future research on the field of strength exercise programs focused on specific body areas in older populations.

There are some limitations that need to be considered when interpreting our findings. First, one of the experimental groups showed significant differences in age with the other experimental and control groups, which is of particular importance when interpreting and comparing the evolution in the assessments across the groups and the study time. Second, a reduced number of participants showed high rates of adherence during the intervention phases finished all assessments. Third, due to the small simple size and heterogeneity on the cognitive status of the participants, it was not possible to perform a stratified analysis taking into account cognitive function. Fourth, the lack of randomization of the sample. Finally, residual potential confounding derived from the lack of a better control of the activities performed by the participants in their daily lives, particularly within the detraining periods, and other unaccounted confounders that could interfere with the results.

In conclusion, in this preliminary study, resistance exercise with elastic bands showed beneficial effects on cognitive function and functional independence in institutionalized older adults. While upper body exercises seem to produce acute effects on cognitive function, lower limb exercises showed better results on physical function parameters. More studies are needed to corroborate these findings and assess more precisely the effects of resistance training with elastic bands on physical and cognitive function in institutionalized older adults.

## Supplementary Information

Below is the link to the electronic supplementary material.

Supplementary file1 (DOCX 2549 kb)

## Data Availability

Data are available.
