# Supplementary material for: Upper versus lower body resistance exercise with elastic bands: effects on cognitive and physical function of institutionalized older adults
Source: Eur Geriatr Med. 2022 Feb 12;13(4):907–16. doi: 10.1007/s41999-022-00616-6 (PMC9378322; doi:10.1007/s41999-022-00616-6)
Supplement: Supplementary file 1 — Supplementary file1 (DOCX 2549 kb) [file 41999_2022_616_MOESM1_ESM.docx]

**SUPPLEMENTARY MATERIAL**

**INDEX**

**Supplementary Table 1.** Session examples of upper- and lower-body resistance exercise programs with elastic bands. **(Page 2)**

**Supplementary Table 2.** Session example of the stretching program. **(Page 3)**

**Supplementary Table 3.** Percentage of change and effect size between measurements using intention to treat analysis (G1 n=20, G2 n=29, CG n=19). **(Page 4)**

**Supplementary Table 4.** Results and comparisons across all measurements using per protocol analysis. **(Page 5)**

**Supplementary Table 5.** Moment per group interaction analysis using per protocol analysis. **(Page 6)**

**Supplementary Table 6.** Percentage of change and effect size between measurements using per protocol analysis. **(Page 7)**

**Supplementary Figure 1.** Evolution of the MEC, TMTA and Phototest outcomes across all study measurements by group and for both intention-to-treat and per-protocol analyses. **(Page 8).**

**Supplementary Figure 2.** Evolution of the TUG, CSR, BS, and HG outcomes across all study measurements by group and for both intention-to-treat and per-protocol analyses. **(Page 9).**

**Supplementary Table 1.** Session examples of upper- and lower-body resistance exercise programs with elastic bands.

| **Session phase (duration)** | **Upper-body resistance exercise program** | | **Lower-body resistance exercise program** | |
| --- | --- | --- | --- | --- |
|  | **Exercise** | **Repetitions, sets and rest between sets** | **Exercise** | **Repetitions, sets and rest between sets** |
| Warm-up  (10 minutes) | Sitting joint mobilization |  | Sitting joint mobilization |  |
|  | Ankles | 5 circles per side, 1 set | Ankles | 5 circles per side, 1 set |
|  | Knees/hip | 5 circles per side, 1 set | Knees/hip | 5 circles per side, 1 set |
|  | Shoulders | 5 circles per side, 1 set | Shoulders | 5 circles per side, 1 set |
|  | Elbows | 5 circles per side, 1 set | Elbows | 5 circles per side, 1 set |
|  | Wrists | 5 circles per side, 1 set | Wrists | 5 circles per side, 1 set |
|  | Approximations with elastic band* |  | Approximations with elastic band |  |
|  | Horizontal band pull | 5 repetitions, 1 set | Leg abductions* | 5 repetitions, 1 set |
|  | Anterior shoulder flexions | 5 repetitions, 1 set | Resisted chair squat* | 5 repetitions, 1 set |
|  | Elbow flexions | 5 repetitions, 1 set |  |  |
|  | Rest between exercises (2 minutes) | | Rest between exercises (2 minutes) | |
| Core  (30 minutes) | Horizontal band pull* | 15 repetitions, 2 sets, 1-minute rest | Resisted chair squat* | 15 repetitions, 2 sets, 1-minute rest |
|  | Rest between exercises (2 minutes) | | Rest between exercises (2 minutes) | |
|  | Arm flexion* | 15 repetitions, 2 sets, 1-minute rest | Leg abductions* | 15 repetitions, 2 sets, 1-minute rest |
|  | Rest between exercises (2 minutes) | | Rest between exercises (2 minutes) | |
|  | Lateral shoulder flexion* | 15 repetitions, 2 sets, 1-minute rest | Unilateral leg extension* | 15 repetitions, 2 sets, 1-minute rest |
|  | Rest between exercises (2 minutes) | | Rest between exercises (2 minutes) | |
|  | Arm pull* | 15 repetitions, 2 sets, 1-minute rest | Unilateral plantar flexion* | 15 repetitions, 2 sets, 1-minute rest |
| Cool-down  (5 minutes) | Stretches: |  | Stretches: |  |
|  | Shoulder and scapular girdle | Maintain 15 seconds | Calves (band-assisted)* | Maintain 15 seconds |
|  | Chest (pectoral muscles) | Maintain 15 seconds | Hamstring (band-assisted)* | Maintain 15 seconds |
|  | Biceps | Maintain 15 seconds | Glutes | Maintain 15 seconds |
|  | Triceps | Maintain 15 seconds |  |  |

*Performed with elastic band (yellow colour).

**Supplementary Table 2.** Session example of the stretching program.

| **Session phase (duration)** | **Exercise** | **Characteristics** |
| --- | --- | --- |
| Warm-up  (10 minutes) | Sitting joint mobilization |  |
|  | Ankles | 5 circles per side, 1 set |
|  | Knees/hip | 5 circles per side, 1 set |
|  | Shoulders | 5 circles per side, 1 set |
|  | Elbows | 5 circles per side, 1 set |
|  | Wrists | 5 circles per side, 1 set |
| Core  (30 minutes) | Stretches | No pain, self-perceived tension of 7/10. |
|  | Calves | Maintain 20 seconds per foot |
|  | Hamstring | Maintain 20 seconds per leg |
|  | Hip flexors | Maintain 20 seconds per leg |
|  | Glutes | Maintain 20 seconds per leg |
|  | Hip abductors | Maintain 20 seconds |
|  | Scapular girdle | Maintain 20 seconds |
|  | Chest (pectoral muscles) | Maintain 20 seconds |
|  | Triceps | Maintain 20 seconds per arm |
|  | Biceps | Maintain 20 seconds per arm |
| Cool-down  (5 minutes) | Relaxation: breathing cycles | 5 cycles of 8 seconds  (4 seconds breathing in, 4 breathing out) |

**Supplementary Table 3.** Percentage of change and effect size between measurements using intention to treat analysis (G1 n=20, G2 n=29, CG n=19).

| **Outcome** | **Comparisons between measurements** | | | | | | | | | |
| --- | --- | --- | --- | --- | --- | --- | --- | --- | --- | --- |
|  | **T0 vs T1** | | **T1 vs T2** | | **T2 vs T3** | | **T3 vs T4** | | **T0 vs T4** | |
|  | **Change (%)** | **Cohen’s *d*** | **Change (%)** | **Cohen’s *d*** | **Change (%)** | **Cohen’s *d*** | **Change (%)** | **Cohen’s *d*** | **Change (%)** | **Cohen’s *d*** |
| MEC (score) |  |  |  |  |  |  |  |  |  |  |
| G1 | 15.1 | 0.72 | 0.0 | 0.00 | -5.4 | -0.30 | -5.3 | -0.23 | 3.1 | 0.13 |
| G2 | -0.5 | -0.02 | -2.3 | -0.09 | -1.4 | -0.05 | 4.2 | 0.16 | 0.0 | 0.00 |
| CG | 0.0 | 0.00 | -2.7 | -0.08 | 2.2 | 0.07 | -5.4 | -0.16 | -5.9 | -0.17 |
| TMT-A (seconds) |  |  |  |  |  |  |  |  |  |  |
| G1 | -28.1 | -0.56 | -13.0 | -0.30 | 22.2 | 0.59 | 35.0 | 0.66 | 3.2 | 0.06 |
| G2 | -17.2 | -0.32 | -6.1 | -0.11 | 3.3 | 0.06 | 5.8 | 0.10 | -15.1 | -0.27 |
| CG | -6.2 | -0.14 | -18.5 | -0.43 | -3.7 | -0.09 | 8.0 | 0.18 | -20.5 | -0.53 |
| Phototest (score) |  |  |  |  |  |  |  |  |  |  |
| G1 | 1.5 | 0.06 | 2.1 | 0.09 | 0.3 | 0.01 | -15.4 | -0.45 | -12.0 | -0.32 |
| G2 | -0.1 | 0.00 | 2.1 | 0.06 | 2.8 | 0.07 | 9.3 | 0.28 | 14.6 | 0.40 |
| CG | -4.8 | -0.11 | 10.7 | 0.21 | 5.1 | 0.10 | -3.2 | -0.06 | 7.2 | 0.14 |
| TUG (seconds) |  |  |  |  |  |  |  |  |  |  |
| G1 | 0.6 | 0.01 | -14.2 | -0.34 | 8.5 | 0.21 | -18.3 | -0.56 | -23.5 | -0.57 |
| G2 | 9.7 | 0.11 | -18.3 | -0.26 | 21.0 | 0.37 | -1.3 | -0.02 | 7.1 | 0.10 |
| CG | -22.1 | -0.33 | 11.6 | 0.23 | 3.5 | 0.08 | 8.3 | 0.17 | -2.6 | -0.04 |
| CSR (cm) |  |  |  |  |  |  |  |  |  |  |
| G1 | -22.4 | -0.81 | 28.9 | 1.26 | 46.0 | 1.03 | -5.0 | -0.07 | 50.7 | 1.78 |
| G2 | 7.5 | 0.24 | 16.0 | 0.45 | 26.2 | 0.70 | 30.0 | 0.77 | 59.8 | 2.27 |
| CG | 15.5 | 0.55 | 6.0 | 0.16 | 27.6 | 0.75 | 25.4 | 0.60 | 57.1 | 2.24 |
| BS (cm) |  |  |  |  |  |  |  |  |  |  |
| G1 | 9.2 | 0.20 | 19.5 | 0.48 | 18.3 | 0.45 | 18.3 | 0.46 | 51.2 | 1.35 |
| G2 | -2.7 | -0.07 | 6.1 | 0.15 | 34.8 | 0.92 | 10.8 | 0.26 | 43.9 | 1.40 |
| CG | 1.5 | 0.04 | 6.2 | 0.15 | 35.4 | 0.96 | 18.9 | 0.52 | 51.6 | 1.76 |
| HG (kg) |  |  |  |  |  |  |  |  |  |  |
| G1 | 4.1 | 0.14 | -5.1 | -0.25 | -31.0 | -1.82 | -34.0 | -1.75 | -55.0 | -2.18 |
| G2 | 9.5 | 0.34 | -6.6 | -0.25 | -33.8 | -1.41 | -24.0 | -1.11 | -48.6 | -2.20 |
| CG | -4.6 | -0.14 | -2.7 | -0.09 | -23.4 | -0.86 | -25.9 | -1.08 | -47.3 | -1.79 |

BS: Back Scratch test; CG: Control group; CSR: Chair Sit-and-Reach Test; G1: experimental group 1; G2: Experimental group 2; HG: Hand Grip strength; MEC: Spanish version of the Mini-Mental State-Examination; TMT-A: Trail Making Test part A; TUG: Timed Up and Go Test. Measurements are T0: Baseline, T1: After first exercise program, T2: after wash-out period; T3: after second exercise program, T4: after the end of follow-up.

**Supplementary Table 4.** Results and comparisons across all measurements using per protocol analysis.

| **Outcome** | **Comparisons between measurements (mean ± SD)** | | | | | | | | | |
| --- | --- | --- | --- | --- | --- | --- | --- | --- | --- | --- |
|  | **T0 vs T1**  **(G1 n=13, G2 n=21, CG n=12)** | | **T1 vs T2**  **(G1 n=12, G2 n=20, CG n=11)** | | **T2 vs T3**  **(G1 n=9, G2 n=15, CG n=7)** | | **T3 vs T4**  **(G1 n=9, G2 n=15, CG n=6)** | | **T0 vs T4**  **(G1 n=9, G2 n=15, CG n=6)** | |
|  | **T0** | **T1** | **T1** | **T2** | **T2** | ***T3*** | ***T3*** | ***T4*** | ***T0*** | ***T4*** |
| MEC (score) |  |  |  |  |  |  |  |  |  |  |
| G1 | 19.3 ± 4.9 | 22.2 ± 3.9* | 22.1 ± 4.1 | 22.9 ± 3.7 | 23.3 ± 3.7 | 22.4 ± 4.5 | 22.4 ± 4.5 | 21.4 ± 5.9 | 20.0 ± 4.7 | 21.4 ± 5.9 |
| G2 | 22 ± 5.1 | 21.8 ± 5.4 | 21.6 ± 5.5 | 20.8 ± 6.6 | 21.3 ± 5.3 | 21.13 ± 6.6 | 21.1 ± 6.6 | 21.1 ± 6.1 | 22.1 ± 4.7 | 21.1 ± 6.1 |
| CG | 17.0 ± 6.8 | 17.9 ± 5.9 | 18 ± 6.1 | 17.3 ± 6.4 | 16.7 ± 4.8 | 17.3 ± 4.4 | 17 ± 4.7 | 14.8 ± 5.0* | 15.2 ± 5.7 | 14.8 ± 5.0 |
| TMT-A (seconds) |  |  |  |  |  |  |  |  |  |  |
| G1 | 224.8 ± 125.6^a^ | 167.5 ± 77.6*^a^ | 168.3 ± 81^a^ | 153.1 ± 69.4^ab^ | 157.9 ± 80.5^a^ | 198.9 ± 90.7 | 198.9 ± 90.7 | 264.9 ± 193.2 | 230.5 ± 146.7 | 264.9 ± 193.2 |
| G2 | 314.4 ± 184.8 | 263.5 ± 150.4* | 262.7 ± 154.3 | 263.5 ± 156.4^a^ | 264.3 ± 156 | 280.2 ± 163.5 | 280.2 ± 163.5 | 294.7 ± 186.3 | 313 ± 204 | 294.7 ± 186.3 |
| CG | 379.5 ± 141.1^b^ | 352.7 ± 179.3^a^ | 349.7 ± 187.7^a^ | 292.6 ± 135.3^a^ | 324.4 ± 155.7^a^ | 331.1 ± 122.7 | 326.9 ± 133.9 | 347 ± 162.4 | 404.5 ± 169.4 | 347 ± 162.4 |
| Phototest (score) |  |  |  |  |  |  |  |  |  |  |
| G1 | 24.8 ± 6.1^a^ | 26 ± 5.5^a^ | 25.9 ± 5.7 | 26.1 ± 5.2 | 25.3 ± 5.2 | 27.7 ± 5.5^a^ | 27.7 ± 5.5^a^ | 26.6 ± 10.1 | 23 ± 4.7^a^ | 26.6 ± 10.1 |
| G2 | 23.6 ± 9 | 22.7 ± 8.2 | 22.6 ± 8.4 | 23.6 ± 10.7 | 25.7 ± 8.7 | 26.4 ± 9.3^b^ | 26.4 ± 9.3^b^ | 27.4 ± 9.3 | 25.1 ± 7.6^b^ | 27.4 ± 9.3* |
| CG | 16.9 ± 6.7^a^ | 16.6 ± 9.7^a^ | 17.8 ± 9.3 | 18.0 ± 9.9 | 18 ± 7.3 | 17.8 ± 7.5^ab^ | 17.1 ± 7.9 ^ab^ | 17.3 ± 7.7 | 15 ± 3.6^ab^ | 17.3 ± 7.7 |
| TUG (seconds) |  |  |  |  |  |  |  |  |  |  |
| G1 | 23.9 ± 13.2 | 19.9 ± 9.5* | 19.7 ± 9.9 | 19.2 ± 8 | 20.1 ± 9.2 | 18.3 ± 7.4 | 18.3 ± 7.4 | 16 ± 6.7 | 22.8 ± 13.5 | 16.0 ± 6.7 |
| G2 | 18.7 ± 14.6 | 19.2 ± 17 | 15.9 ± 8.1 | 16.8 ± 7.4 | 16.3 ± 7.9 | 24.2 ± 13.3* | 24.2 ± 13.3 | 20.9 ± 15.2 | 16.4 ± 7.0 | 20.9 ± 15.2 |
| CG | 20.2 ± 8.4 | 20.1 ± 10.7 | 20.7 ± 11 | 21.9 ± 7.9* | 24.8 ± 6.6 | 25.6 ± 8.7 | 24.9 ± 9.4 | 24.5 ± 9.1 | 20.8 ± 5.3 | 24.5 ± 9.1 |
| CSR (cm) |  |  |  |  |  |  |  |  |  |  |
| G1 | -30.1 ± 11.1 | -37.6 ± 5*^ab^ | -38.1 ± 4.9^ab^ | -27.7 ± 11.4* | -24.6 ± 11.2 | -18.1 ± 10* | -18.1 ± 10 | -14.0 ± 6.4* | -28.4 ± 12.7 | -14.0 ± 6.4* |
| G2 | -30.6 ± 10 | -28.4 ± 11.5^a^ | -28.9 ± 11.6^a^ | -23.2 ± 12.2* | -22.4 ± 13.9 | -17.1 ± 9.5* | -17.1 ± 9.5 | -12.0 ± 7.4* | -29.8 ± 10.2 | -12.0 ± 7.4* |
| CG | -31.5 ± 7 | -28.7 ± 6.5*^b^ | -29.1 ± 6.6^a^ | -25.2 ± 11 | -28.5 ± 8.6 | -21.1 ± 4.5* | -21.4 ± 4.9 | -18.2 ± 9.4 | -31.5 ± 6.1 | -18.2 ± 9.4* |
| BS (cm) |  |  |  |  |  |  |  |  |  |  |
| G1 | -29.0 ± 12.9 | -27.4 ± 11.3 | -27.2 ± 11.8 | -23.5 ± 9.0* | -25.8 ± 8.2 | -19.5 ± 9.5* | -19.5 ± 9.5 | -14.1 ± 4.6* | -31.1 ± 12.6 | -14.1 ± 4.6* |
| G2 | -31.6 ± 10.4 | -33.3 ± 11.5 | -33.3 ± 11.8 | -31.6 ± 10.9 | -31.3 ± 11.6 | -21.1 ± 9.7* | -21.1 ± 9.7 | -17.6p ± 6.1* | -30.5 ± 10.4 | -17.6 ± 6.1* |
| CG | -34.0 ± 11 | -36.0 ± 13.8 | -33.7 ± 11.7 | -31.4 ± 12.7 | -33.7 ± 12.7 | -22.2 ± 9.3* | -24.2 ± 8.5 | -19.6 ± 4.2 | -33.3 ± 10.1 | -19.6 ± 4.2* |
| HG (kg) |  |  |  |  |  |  |  |  |  |  |
| G1 | 17.9 ± 4.5 | 18.9 ± 3.5^a^ | 19.2 ± 3.4^a^ | 18.4 ± 4.0 | 18.5 ± 4.5 | 13.0 ± 3.9* | 13.0 ± 3.9 | 9.0 ± 3.0* | 18.7 ± 5.1 | 9.0 ± 3.0* |
| G2 | 20.3 ± 5.3 | 22.6 ± 5.0*^ab^ | 22.6 ± 5.1^a^ | 20.1 ± 6.0* | 18.9 ± 5.0 | 13.4 ± 3.5* | 13.4 ± 3.5 | 10.4 ± 2.5* | 18.7 ± 4.9 | 10.4 ± 2.5* |
| CG | 23.9 ± 13.2 | 18 ± 5.8^b^ | 18.4 ± 5.9 | 18.3 ± 6.7 | 16.4 ± 6.3 | 12.7 ± 5.8 | 13.4 ± 6.1 | 9 ± 2.8* | 17.8 ± 6.6 | 9 ± 2.8* |

*Significant differences (p<0.05) with previous moment of assessment for the same group; ^a^ or ^b^: Significant differences between groups with the same upper script letter in the moment of assessment.

BS: Back Scratch test; CG: Control group; CSR: Chair Sit-and-Reach Test; G1: experimental group 1; G2: Experimental group 2; HG: Hand Grip strength; MEC: Spanish version of the Mini-Mental State-Examination; TMT-A: Trail Making Test part A; TUG: Timed Up and Go Test. Measurements are T0: Baseline, T1: After first exercise program, T2: after wash-out period; T3: after second exercise program, T4: after the end of follow-up.

**Supplementary Table 5.** Moment per group interaction analysis using per protocol analysis.

| **Outcome** | **Moment x group interactions** | | | | | |
| --- | --- | --- | --- | --- | --- | --- |
|  | **T0 vs T1**  **(G1 n=13, G2 n=21, CG n=12)** | | **T2 vs T3**  **(G1 n=9, G2 n=15, CG n=7)** | | **T0 vs T4**  **(G1 n=9, G2 n=15, CG n=6)** | |
|  | ***F* (2, 86)** | ***P*** | ***F* (2, 56)** | ***P*** | ***F* (8, 135)** | ***P*** |
| MEC | 0.67 | 0.513 | 0.08 | 0.919 | 0.29 | 0.968 |
| TMT-A | 0.07 | 0.931 | 0.07 | 0.931 | 0.15 | 0.996 |
| Phototest | 0.13 | 0.878 | 0.12 | 0.885 | 0.22 | 0.988 |
| TUG | 0.24 | 0.790 | 1.59 | 0.214 | 0.92 | 0.502 |
| CSR | 2.66 | 0.076 | 0.05 | 0.952 | 0.80 | 0.603 |
| BS | 0.20 | 0.821 | 0.30 | 0.743 | 0.20 | 0.990 |
| HG | 0.93 | 0.400 | 0.21 | 0.814 | 0.37 | 0.935 |

BS: Back Scratch test; CG: Control group; CSR: Chair Sit-and-Reach Test; G1: experimental group 1; G2: Experimental group 2; HG: Hand Grip strength; MEC: Spanish version of the Mini-Mental State-Examination; TMT-A: Trail Making Test part A; TUG: Timed Up and Go Test. Measurements are T0: Baseline, T1: After first exercise program, T2: after wash-out period; T3: after second exercise program, T4: after the end of follow-up.

**Supplementary Table 6.** Percentage of change and effect size between measurements using per protocol analysis.

| **Outcome** | **Comparisons between measurements** | | | | | | | | | |
| --- | --- | --- | --- | --- | --- | --- | --- | --- | --- | --- |
|  | **T0 vs T1**  **(G1 n=13, G2 n=21, CG n=12)** | | **T1 vs T2**  **(G1 n=12, G2 n=20, CG n=11)** | | **T2 vs T3**  **(G1 n=9, G2 n=15, CG n=7)** | | **T3 vs T4**  **(G1 n=9, G2 n=15, CG n=6)** | | **T0 vs T4**  **(G1 n=9, G2 n=15, CG n=6)** | |
|  | **Change (%)** | **Hedges’ *g*** | **Change (%)** | **Hedges’ *g*** | **Change (%)** | **Hedges’ *g*** | **Change (%)** | **Hedges’ *g*** | **Change (%)** | **Hedges’ *g*** |
| MEC (score) |  |  |  |  |  |  |  |  |  |  |
| G1 | 15.0 | 0.63 | 3.6 | 0.20 | -3.9 | -0.21 | -4.5 | -0.18 | 7.0 | 0.25 |
| G2 | -0.9 | -0.04 | -3.7 | -0.13 | -0.8 | -0.03 | 0.0 | 0.00 | -4.5 | -0.18 |
| CG | 5.3 | 0.14 | -3.9 | -0.11 | 3.6 | 0.12 | -12.9 | -0.42 | -2.6 | -0.07 |
| TMT-A (seconds) |  |  |  |  |  |  |  |  |  |  |
| G1 | -25.5 | -0.53 | -9.0 | -0.19 | 26.0 | 0.45 | 33.2 | 0.42 | 14.9 | 0.19 |
| G2 | -16.2 | -0.30 | 0.3 | 0.00 | 6.0 | 0.10 | 5.2 | 0.08 | -5.8 | -0.09 |
| CG | -7.1 | -0.16 | -16.3 | -0.34 | 2.1 | 0.04 | 6.1 | 0.12 | -14.2 | -0.32 |
| Phototest (score) |  |  |  |  |  |  |  |  |  |  |
| G1 | 4.8 | 0.19 | 0.8 | 0.04 | 9.5 | 0.43 | -4.0 | -0.13 | 15.7 | 0.44 |
| G2 | -3.8 | -0.10 | 4.4 | 0.10 | 2.7 | 0.08 | 3.8 | 0.10 | 9.2 | 0.27 |
| CG | -1.8 | -0.03 | 1.1 | 0.02 | -1.1 | 0.02 | 1.2 | 0.02 | 15.3 | 0.35 |
| TUG (seconds) |  |  |  |  |  |  |  |  |  |  |
| G1 | -16.7 | -0.33 | -2.5 | -0.06 | -9.0 | -0.21 | -12.6 | -0.30 | -29.8 | -0.60 |
| G2 | 2.7 | 0.03 | 5.7 | 0.11 | 48.5 | 0.70 | -13.6 | -0.22 | 27.4 | 0.37 |
| CG | -0.5 | -0.01 | 5.8 | 0.12 | 3.2 | 0.10 | -1.6 | -0.04 | 17.8 | 0.46 |
| CSR (cm) |  |  |  |  |  |  |  |  |  |  |
| G1 | -24.9 | -0.84 | 27.3 | 1.14 | 26.4 | 0.57 | 22.7 | 0.46 | 50.7 | 1.35 |
| G2 | 7.2 | 0.20 | 19.7 | 0.46 | 23.7 | 0.43 | 29.8 | 0.57 | 59.7 | 1.93 |
| CG | 8.9 | 0.40 | 13.4 | 0.41 | 26.0 | 1.00 | 15.0 | 0.39 | 42.2 | 1.54 |
| BS (cm) |  |  |  |  |  |  |  |  |  |  |
| G1 | 5.5 | 0.13 | 13.6 | 0.34 | 24.4 | 0.67 | 27.7 | 0.69 | 54.7 | 1.70 |
| G2 | -5.4 | -0.15 | 5.1 | 0.15 | 32.6 | 0.92 | 16.6 | 0.41 | 42.3 | 1.46 |
| CG | -5.9 | -0.16 | 6.8 | 0.17 | 34.1 | 0.97 | 19.0 | 0.63 | 41.1 | 1.63 |
| HG (kg) |  |  |  |  |  |  |  |  |  |  |
| G1 | 5.6 | 0.24 | -4.2 | -0.21 | -29.7 | -1.23 | -30.8 | -1.09 | -51.9 | -2.20 |
| G2 | 11.3 | 0.44 | -11.1 | -0.43 | -29.1 | -1.23 | -22.4 | -0.95 | -44.4 | -2.05 |
| CG | -7.2 | -0.20 | -0.5 | -0.02 | -22.6 | -0.56 | -32.8 | -0.86 | -49.4 | -1.59 |

BS: Back Scratch test; CG: Control group; CSR: Chair Sit-and-Reach Test; G1: experimental group 1; G2: Experimental group 2; HG: Hand Grip strength; MEC: Spanish version of the Mini-Mental State-Examination; TMT-A: Trail Making Test part A; TUG: Timed Up and Go Test. Measurements are T0: Baseline, T1: After first exercise program, T2: after wash-out period; T3: after second exercise program, T4: after the end of follow-up.

**
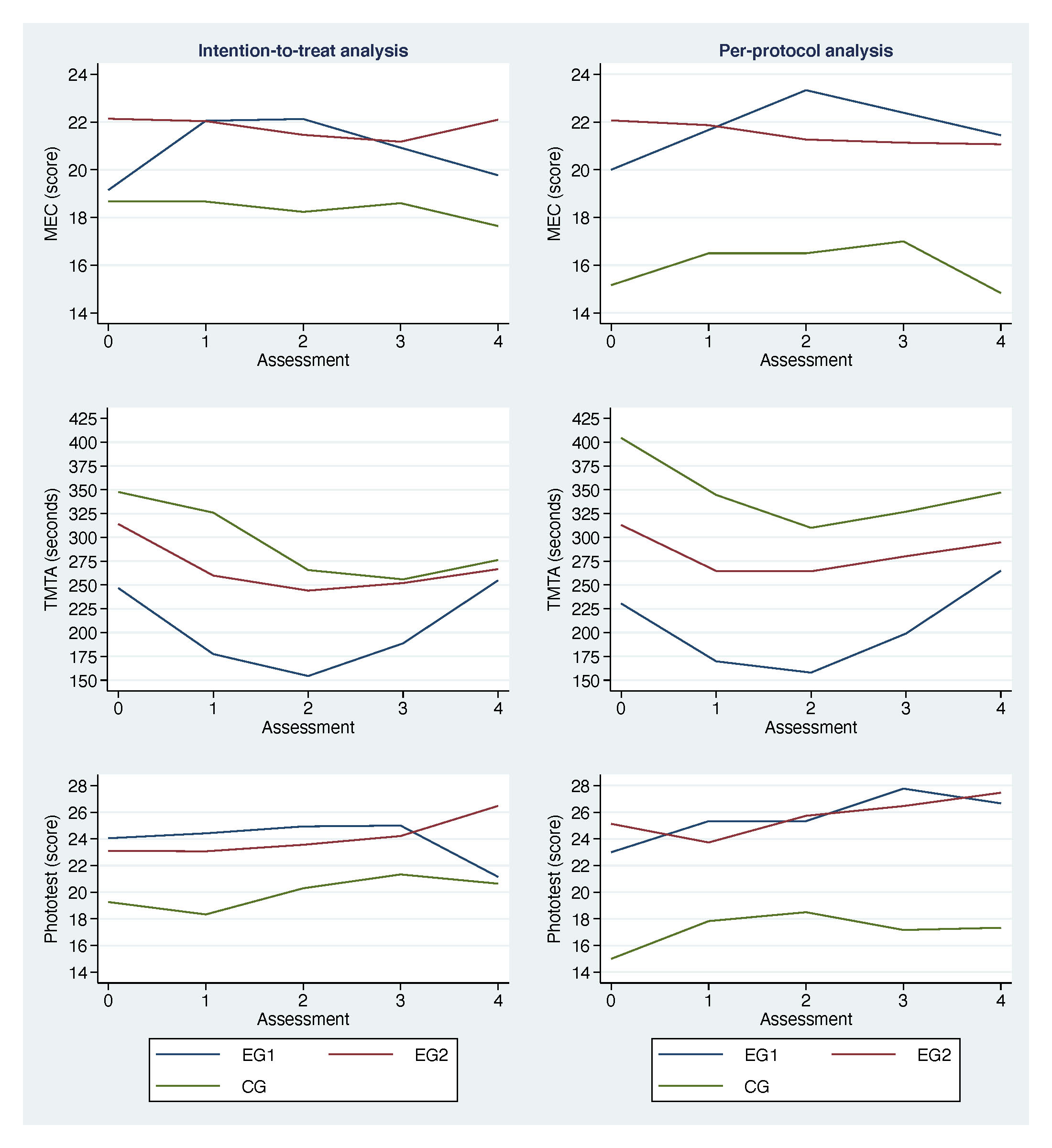
**

**Supplementary Figure 1.** Evolution of the Spanish version of the Mini-Mental State-Examination (MEC), Trail Making Test Part A (TMTA) and Phototest outcomes across all study measurements by group and for both intention-to-treat and per-protocol analyses. EG1: Experimental group 1; EG2: experimental group 2; CG: control group.

**
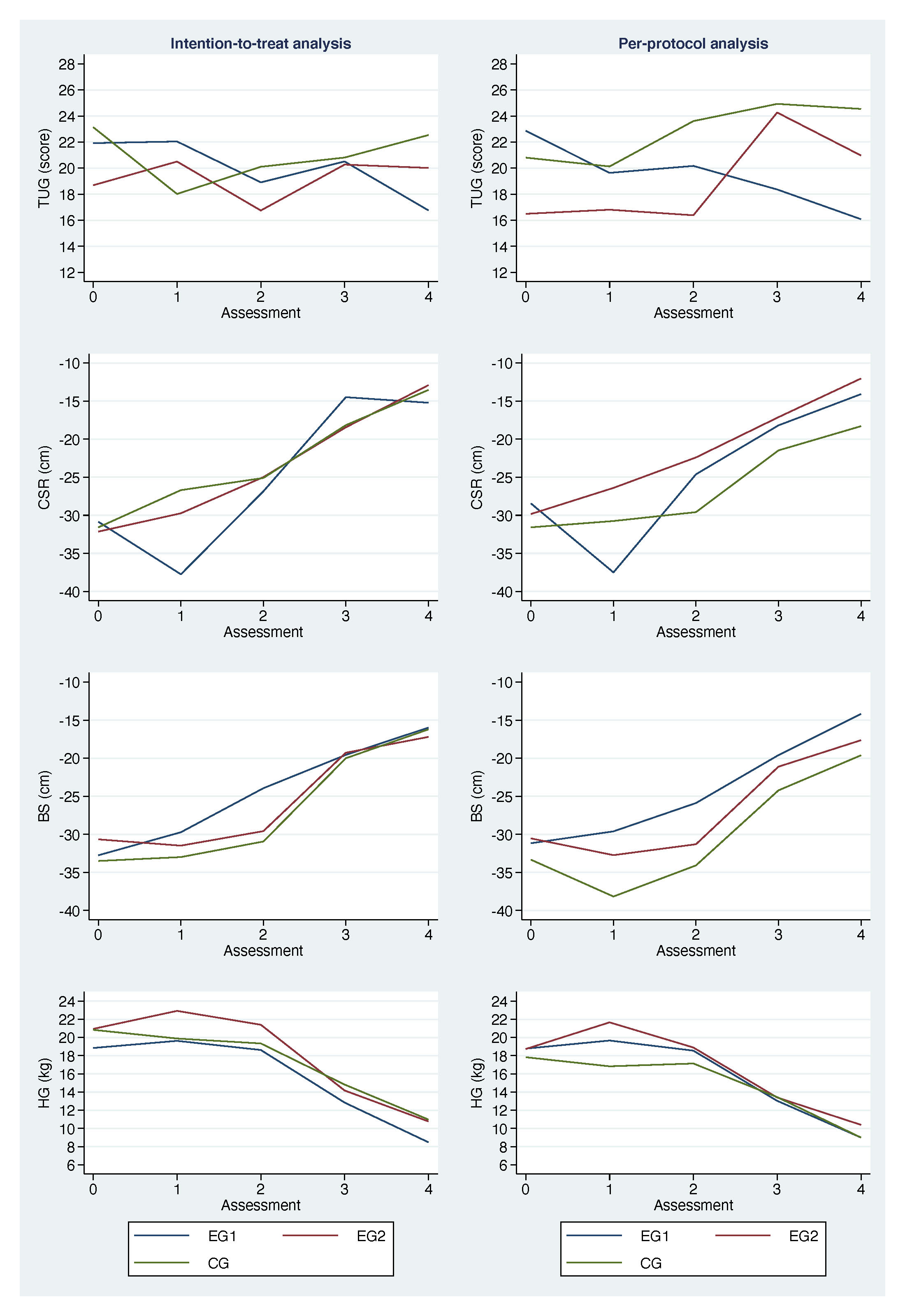
**

**Supplementary Figure 2.** Evolution of the Timed Up and Go Test (TUG), Chair Sit-and-reach (CSR), Back Scratch (BS), and hand grip strength (HG) outcomes across all study measurements by group and for both intention-to-treat and per-protocol analyses. EG1: Experimental group 1; EG2: experimental group 2; CG: control group.
